# Supplementary material for: A Likelihood Approach for Real-Time Calibration of Stochastic Compartmental Epidemic Models
Source: PLoS Comput Biol. 2017 Jan 17;13(1):e1005257. doi: 10.1371/journal.pcbi.1005257 (PMC5240920; doi:10.1371/journal.pcbi.1005257)
Supplement: S1 File — (TAR.GZ) [file pcbi.1005257.s014.tar.gz › HSPH_Online-SI-Revision/PF08/n10-severe/n10-severe_PF2_table0.pdf]

1	newly 3 weeks spec	422.	427.5	{387., 462.}	5.90995	0.0255419	newly 3 weeks cumul	1572.	1352.85	{1260., 1437.}	13.9408	0.0659376	newly 5 weeks spec	319.	346.25	{308., 381.}	10.906	0.0448487	newly 5 weeks cumul	2230.	2084.76	{1955., 2190.}	6.56771	0.0301579	newly 51 weeks cumul	3236.	4103.9	{3699., 4506.}	27.186	0.10348
2	newly 3 weeks spec	559.	535.97	{495., 584.}	6.36673	0.0289458	newly 3 weeks cumul	1850.	1708.13	{1609., 1812.}	7.74541	0.0357413	newly 5 weeks spec	307.	443.82	{396., 498.}	44.6971	0.158639	newly 5 weeks cumul	2596.	2652.09	{2499., 2824.}	4.82242	0.0207416	newly 53 weeks cumul	3551.	4955.13	{4506., 5481.}	39.5418	0.143158
3	newly 3 weeks spec	372.	587.71	{522., 660.}	57.9866	0.196605	newly 3 weeks cumul	1462.	1891.91	{1697., 2069.}	29.4056	0.110529	newly 5 weeks spec	176.	465.4	{401., 527.}	164.432	0.419577	newly 5 weeks cumul	1920.	2890.7	{2599., 3170.}	50.5573	0.176268	newly 55 weeks cumul	2258.	5087.65	{4558., 5616.}	125.317	0.350625
4	newly 3 weeks spec	431.	397.34	{365., 430.}	8.47332	0.0392066	newly 3 weeks cumul	1668.	1261.09	{1156., 1321.}	24.3951	0.122034	newly 5 weeks spec	272.	328.99	{293., 362.}	21.1949	0.0820978	newly 5 weeks cumul	2316.	1951.04	{1814., 2058.}	15.7582	0.0751006	newly 50 weeks cumul	2990.	3981.74	{3644., 4193.}	33.1926	0.1237
5	newly 3 weeks spec	395.	401.65	{346., 441.}	7.43038	0.03216	newly 3 weeks cumul	1512.	1273.7	{1081., 1362.}	15.7606	0.0761027	newly 5 weeks spec	274.	334.92	{295., 375.}	22.2628	0.0857125	newly 5 weeks cumul	2125.	1978.87	{1743., 2122.}	7.38965	0.0342782	newly 50 weeks cumul	3018.	4067.81	{3792., 4306.}	34.785	0.129146
6	newly 3 weeks spec	500.	409.13	{375., 445.}	18.174	0.0880859	newly 3 weeks cumul	1731.	1272.55	{1206., 1336.}	26.4847	0.134052	newly 5 weeks spec	216.	352.41	{319., 388.}	63.1528	0.211303	newly 5 weeks cumul	2283.	2009.81	{1899., 2111.}	11.9663	0.0557448	newly 51 weeks cumul	2710.	4348.53	{4071., 4591.}	60.4624	0.204891
7	newly 3 weeks spec	266.	367.55	{317., 413.}	38.2068	0.138028	newly 3 weeks cumul	943.	1168.81	{1006., 1309.}	23.982	0.0912032	newly 5 weeks spec	142.	305.25	{267., 343.}	114.965	0.330088	newly 5 weeks cumul	1279.	1813.52	{1599., 2009.}	41.792	0.149785	newly 50 weeks cumul	1805.	3790.57	{3422., 4201.}	110.004	0.320066
8	newly 3 weeks spec	348.	586.27	{539., 631.}	68.4684	0.225735	newly 3 weeks cumul	1582.	1876.76	{1743., 2010.}	18.6384	0.073582	newly 5 weeks spec	203.	460.21	{426., 496.}	126.704	0.35466	newly 5 weeks cumul	2074.	2867.63	{2688., 3003.}	38.2657	0.140311	newly 54 weeks cumul	2481.	5079.83	{4862., 5280.}	104.749	0.310969
9	newly 3 weeks spec	571.	799.6	{664., 940.}	40.0595	0.142733	newly 3 weeks cumul	2047.	2489.85	{2064., 2873.}	22.3415	0.0850992	newly 5 weeks spec	361.	607.46	{499., 696.}	68.2715	0.222152	newly 5 weeks cumul	2908.	3809.68	{3186., 4347.}	31.1582	0.115037	newly 58 weeks cumul	3573.	6225.25	{5205., 6870.}	74.2303	0.237769
10	newly 3 weeks spec	479.	686.47	{613., 759.}	43.3132	0.154617	newly 3 weeks cumul	1759.	2343.88	{2119., 2603.}	33.2507	0.123183	newly 5 weeks spec	209.	467.52	{415., 533.}	123.694	0.347361	newly 5 weeks cumul	2339.	3385.58	{3112., 3754.}	44.7448	0.159297	newly 58 weeks cumul	2666.	5052.39	{4625., 5586.}	89.512	0.276151
11	newly 3 weeks spec	358.	784.2	{711., 844.}	119.05	0.339457	newly 3 weeks cumul	1919.	2482.81	{2301., 2662.}	29.3804	0.111014	newly 5 weeks spec	90.	590.58	{536., 640.}	556.2	0.815907	newly 5 weeks cumul	2152.	3762.18	{3495., 4003.}	74.8225	0.241883	newly 57 weeks cumul	2254.	6154.13	{5826., 6411.}	173.031	0.435658
12	newly 3 weeks spec	587.	762.03	{672., 821.}	29.8722	0.112237	newly 3 weeks cumul	1997.	2318.34	{2066., 2480.}	16.9975	0.0676653	newly 5 weeks spec	284.	611.62	{547., 687.}	115.359	0.331706	newly 5 weeks cumul	2683.	3622.58	{3268., 3859.}	35.0198	0.129345	newly 57 weeks cumul	3203.	6285.28	{5790., 6662.}	96.231	0.291973
13	newly 3 weeks spec	574.	580.53	{536., 616.}	4.42683	0.0190664	newly 3 weeks cumul	2148.	1855.01	{1753., 1964.}	13.6401	0.0641072	newly 5 weeks spec	276.	457.09	{413., 497.}	65.6123	0.218067	newly 5 weeks cumul	2826.	2834.63	{2697., 2961.}	3.32449	0.0144634	newly 54 weeks cumul	3484.	5031.87	{4707., 5223.}	44.428	0.159287
14	newly 3 weeks spec	353.	662.98	{613., 712.}	87.813	0.272792	newly 3 weeks cumul	1439.	2193.74	{2012., 2308.}	52.4489	0.182406	newly 5 weeks spec	163.	479.78	{433., 524.}	194.344	0.467587	newly 5 weeks cumul	1856.	3243.14	{2961., 3395.}	74.7381	0.241751	newly 56 weeks cumul	2090.	5163.59	{4835., 5494.}	147.062	0.392048
15	newly 3 weeks spec	511.	910.17	{818., 998.}	78.1155	0.249384	newly 3 weeks cumul	2278.	3120.31	{2871., 3379.}	36.9759	0.135723	newly 5 weeks spec	197.	585.21	{523., 652.}	197.061	0.471169	newly 5 weeks cumul	2829.	4451.4	{4097., 4812.}	57.3489	0.195947	newly 60 weeks cumul	3055.	6260.21	{5684., 6800.}	104.917	0.310458
16	newly 3 weeks spec	366.	623.54	{516., 712.}	70.3661	0.228278	newly 3 weeks cumul	1694.	2183.85	{1949., 2412.}	28.9168	0.1086	newly 5 weeks spec	184.	421.51	{311., 493.}	129.082	0.353086	newly 5 weeks cumul	2137.	3121.21	{2672., 3432.}	46.0557	0.162201	newly 56 weeks cumul	2454.	4625.84	{3452., 5316.}	88.502	0.270188
17	newly 3 weeks spec	538.	597.22	{517., 675.}	13.0446	0.0525513	newly 3 weeks cumul	1689.	1813.72	{1589., 2050.}	10.0438	0.0413768	newly 5 weeks spec	353.	508.01	{440., 583.}	43.9122	0.155537	newly 5 weeks cumul	2470.	2880.86	{2547., 3253.}	17.6721	0.0697094	newly 56 weeks cumul	3959.	5551.41	{4713., 6118.}	40.2225	0.145093
18	newly 3 weeks spec	567.	528.69	{452., 591.}	8.49912	0.039742	newly 3 weeks cumul	1753.	1666.71	{1441., 1806.}	6.66115	0.0308357	newly 5 weeks spec	394.	441.33	{396., 483.}	13.7284	0.055598	newly 5 weeks cumul	2605.	2599.21	{2279., 2788.}	5.69021	0.025361	newly 54 weeks cumul	3791.	4894.68	{4471., 5298.}	29.6149	0.111509
19	newly 3 weeks spec	442.	430.01	{393., 463.}	5.50905	0.0246395	newly 3 weeks cumul	1574.	1383.12	{1322., 1441.}	12.1271	0.0565048	newly 5 weeks spec	313.	347.79	{319., 375.}	11.639	0.0472121	newly 5 weeks cumul	2213.	2117.48	{1998., 2199.}	4.42115	0.0199481	newly 50 weeks cumul	2944.	4097.99	{3881., 4303.}	39.198	0.143269
20	newly 3 weeks spec	441.	319.65	{283., 359.}	27.517	0.14165	newly 3 weeks cumul	1300.	991.12	{895., 1108.}	23.76	0.1192	newly 5 weeks spec	373.	284.69	{253., 314.}	23.6756	0.119089	newly 5 weeks cumul	2074.	1579.86	{1440., 1738.}	23.8255	0.11933	newly 50 weeks cumul	3655.	3827.78	{3607., 4081.}	5.8922	0.0246786
21	newly 3 weeks spec	460.	728.47	{650., 811.}	58.363	0.197958	newly 3 weeks cumul	1920.	2432.78	{2149., 2697.}	26.7073	0.101165	newly 5 weeks spec	202.	508.26	{436., 557.}	151.614	0.398369	newly 5 weeks cumul	2437.	3556.05	{3168., 3940.}	45.9192	0.16266	newly 58 weeks cumul	2952.	5368.03	{4898., 6083.}	81.8438	0.257956
22	newly 3 weeks spec	509.	565.02	{501., 627.}	12.2908	0.0492685	newly 3 weeks cumul	1513.	1701.04	{1533., 1837.}	12.842	0.0513779	newly 5 weeks spec	221.	497.69	{443., 561.}	125.199	0.350599	newly 5 weeks cumul	2121.	2742.49	{2496., 2973.}	29.3017	0.11037	newly 56 weeks cumul	2643.	5558.78	{4941., 6139.}	110.321	0.321287
23	newly 3 weeks spec	421.	534.46	{437., 601.}	28.2328	0.10621	newly 3 weeks cumul	1389.	1681.61	{1421., 1843.}	22.1879	0.0854976	newly 5 weeks spec	276.	439.22	{361., 503.}	59.6812	0.199334	newly 5 weeks cumul	2005.	2606.72	{2210., 2888.}	30.8738	0.114993	newly 55 weeks cumul	2915.	4857.43	{3966., 5482.}	67.0453	0.218799
24	newly 3 weeks spec	587.	726.62	{676., 782.}	23.7853	0.0919979	newly 3 weeks cumul	2481.	2303.63	{2230., 2389.}	7.14913	0.0324997	newly 5 weeks spec	276.	557.65	{507., 616.}	102.047	0.304216	newly 5 weeks cumul	3169.	3510.51	{3345., 3664.}	10.7766	0.0441533	newly 56 weeks cumul	3630.	5927.92	{5451., 6337.}	63.3036	0.212168
25	newly 3 weeks spec	500.	952.2	{889., 1013.}	90.44	0.279261	newly 3 weeks cumul	2503.	3032.03	{2913., 3154.}	21.1358	0.0830252	newly 5 weeks spec	93.	676.62	{606., 736.}	627.548	0.860773	newly 5 weeks cumul	2800.	4526.77	{4347., 4706.}	61.6704	0.208352	newly 59 weeks cumul	2905.	6914.4	{6643., 7188.}	138.017	0.376258
26	newly 3 weeks spec	372.	669.61	{553., 773.}	80.0027	0.251664	newly 3 weeks cumul	1508.	2113.42	{1772., 2400.}	40.183	0.14385	newly 5 weeks spec	264.	511.83	{397., 615.}	93.875	0.282521	newly 5 weeks cumul	2071.	3222.23	{2727., 3636.}	55.5881	0.189094	newly 58 weeks cumul	2508.	5286.81	{4221., 6457.}	110.798	0.318668
27	newly 3 weeks spec	638.	856.62	{709., 966.}	34.721	0.126923	newly 3 weeks cumul	2129.	2643.39	{2241., 2933.}	24.7163	0.0940032	newly 5 weeks spec	370.	648.88	{546., 746.}	75.373	0.240348	newly 5 weeks cumul	2994.	4048.75	{3454., 4515.}	35.4906	0.129748	newly 59 weeks cumul	3855.	6517.73	{5820., 7206.}	69.0721	0.225438
28	newly 3 weeks spec	519.	844.26	{770., 909.}	62.6705	0.210259	newly 3 weeks cumul	1948.	2655.29	{2375., 2939.}	36.3085	0.13324	newly 5 weeks spec	219.	631.34	{565., 682.}	188.283	0.458554	newly 5 weeks cumul	2468.	4026.96	{3630., 4335.}	63.1669	0.211761	newly 58 weeks cumul	2735.	6488.94	{6069., 6885.}	137.256	0.3746
29	newly 3 weeks spec	430.	932.68	{863., 999.}	116.902	0.33553	newly 3 weeks cumul	2046.	3151.32	{2902., 3333.}	54.0235	0.187099	newly 5 weeks spec	157.	611.02	{548., 665.}	289.185	0.588832	newly 5 weeks cumul	2479.	4529.5	{4166., 4810.}	82.7148	0.261293	newly 60 weeks cumul	2601.	6449.41	{5966., 6853.}	147.959	0.393739
30	newly 3 weeks spec	581.	993.58	{920., 1054.}	71.012	0.23245	newly 3 weeks cumul	2678.	3330.22	{3133., 3486.}	24.3547	0.0942997	newly 5 weeks spec	176.	650.37	{599., 702.}	269.528	0.566832	newly 5 weeks cumul	3250.	4801.25	{4554., 5027.}	47.7308	0.169122	newly 60 weeks cumul	3417.	6875.06	{6485., 7249.}	101.202	0.30325
31	newly 3 weeks spec	369.	583.1	{450., 701.}	58.1192	0.193888	newly 3 weeks cumul	1285.	1867.37	{1501., 2179.}	45.3206	0.158252	newly 5 weeks spec	236.	446.75	{333., 537.}	89.5805	0.271422	newly 5 weeks cumul	1779.	2828.79	{2188., 3305.}	59.0101	0.196991	newly 57 weeks cumul	2329.	4794.42	{3672., 5780.}	105.894	0.307263
32	newly 3 weeks spec	297.	327.44	{282., 369.}	12.3973	0.049939	newly 3 weeks cumul	969.	1038.86	{914., 1175.}	9.67183	0.039611	newly 5 weeks spec	208.	274.69	{241., 313.}	32.3413	0.119527	newly 5 weeks cumul	1426.	1615.12	{1430., 1778.}	13.8822	0.0552304	newly 50 weeks cumul	2189.	3506.28	{3122., 3867.}	60.1772	0.202362
33	newly 3 weeks spec	452.	369.	{324., 414.}	18.3628	0.089921	newly 3 weeks cumul	1482.	1156.57	{1011., 1265.}	21.9588	0.109253	newly 5 weeks spec	292.	315.21	{276., 350.}	10.3801	0.0426141	newly 5 weeks cumul	2155.	1815.27	{1591., 1981.}	15.7647	0.0758068	newly 50 weeks cumul	3095.	3975.53	{3637., 4288.}	28.4501	0.107886
34	newly 3 weeks spec	392.	383.89	{329., 444.}	8.23724	0.0367115	newly 3 weeks cumul	1312.	1231.14	{1077., 1327.}	8.3872	0.0385052	newly 5 weeks spec	229.	310.06	{261., 353.}	35.5808	0.12932	newly 5 weeks cumul	1839.	1888.72	{1619., 2110.}	7.65307	0.0327155	newly 50 weeks cumul	2740.	3801.67	{3354., 4190.}	39.1675	0.14174
35	newly 3 weeks spec	441.	717.75	{637., 777.}	62.7551	0.209978	newly 3 weeks cumul	1805.	2261.53	{1982., 2431.}	25.462	0.0974208	newly 5 weeks spec	239.	559.09	{499., 606.}	133.929	0.36726	newly 5 weeks cumul	2353.	3467.46	{3088., 3685.}	47.3634	0.167266	newly 57 weeks cumul	2899.	5847.59	{5277., 6313.}	101.711	0.303434
36	newly 3 weeks spec	507.	509.3	{429., 569.}	8.39053	0.0367826	newly 3 weeks cumul	1570.	1575.33	{1364., 1748.}	7.29745	0.0319861	newly 5 weeks spec	332.	434.89	{364., 493.}	31.762	0.117676	newly 5 weeks cumul	2346.	2487.68	{2125., 2770.}	9.66496	0.0403649	newly 53 weeks cumul	3516.	4998.98	{4313., 5537.}	42.318	0.151106
37	newly 3 weeks spec	390.	592.56	{507., 676.}	51.9436	0.178566	newly 3 weeks cumul	1418.	1807.89	{1571., 2047.}	27.8893	0.104988	newly 5 weeks spec	278.	491.78	{425., 574.}	76.8993	0.243977	newly 5 weeks cumul	2082.	2851.93	{2491., 3232.}	37.256	0.135575	newly 56 weeks cumul	2812.	5411.84	{4660., 6044.}	92.4552	0.281083
38	newly 3 weeks spec	545.	659.04	{591., 718.}	21.2514	0.0825813	newly 3 weeks cumul	1921.	2073.91	{1860., 2268.}	9.73712	0.0398798	newly 5 weeks spec	367.	530.47	{473., 578.}	44.5422	0.158358	newly 5 weeks cumul	2763.	3207.37	{2936., 3446.}	16.5983	0.0659275	newly 56 weeks cumul	3774.	5668.39	{5230., 6111.}	50.1958	0.175659
39	newly 3 weeks spec	417.	457.51	{392., 523.}	13.1823	0.0538714	newly 3 weeks cumul	1435.	1437.81	{1226., 1634.}	8.42997	0.0373621	newly 5 weeks spec	292.	384.14	{312., 441.}	32.0205	0.117673	newly 5 weeks cumul	2099.	2245.48	{1867., 2547.}	11.3006	0.0471723	newly 52 weeks cumul	3353.	4526.4	{3558., 5061.}	35.5431	0.129544
40	newly 3 weeks spec	501.	480.72	{393., 533.}	7.49301	0.0349468	newly 3 weeks cumul	1605.	1520.15	{1340., 1673.}	7.2243	0.0337461	newly 5 weeks spec	314.	398.06	{350., 442.}	27.3376	0.103589	newly 5 weeks cumul	2336.	2360.19	{2104., 2580.}	7.0869	0.0311601	newly 52 weeks cumul	3452.	4644.3	{4247., 4946.}	35.0301	0.12966
41	newly 3 weeks spec	586.	922.5	{863., 983.}	57.4232	0.196424	newly 3 weeks cumul	2370.	2980.61	{2833., 3214.}	25.7641	0.0990197	newly 5 weeks spec	259.	646.78	{598., 697.}	149.722	0.39651	newly 5 weeks cumul	2995.	4415.21	{4209., 4713.}	47.4194	0.168135	newly 60 weeks cumul	3338.	6627.39	{6306., 6962.}	98.5437	0.297444
42	newly 3 weeks spec	574.	473.61	{436., 512.}	17.4895	0.0843987	newly 3 weeks cumul	2136.	1486.53	{1408., 1556.}	30.4059	0.157749	newly 5 weeks spec	240.	393.75	{355., 431.}	64.0625	0.213893	newly 5 weeks cumul	2747.	2316.17	{2218., 2404.}	15.6837	0.074385	newly 51 weeks cumul	3203.	4601.41	{4334., 4948.}	43.6594	0.156628
43	newly 3 weeks spec	451.	879.72	{809., 956.}	95.0599	0.289306	newly 3 weeks cumul	1975.	2940.95	{2699., 3123.}	48.9089	0.172282	newly 5 weeks spec	196.	591.63	{533., 654.}	201.852	0.478631	newly 5 weeks cumul	2487.	4261.7	{3932., 4528.}	71.3591	0.23339	newly 59 weeks cumul	2787.	6228.71	{5854., 6776.}	123.492	0.348627
44	newly 3 weeks spec	403.	497.7	{425., 552.}	23.8412	0.091169	newly 3 weeks cumul	1374.	1552.79	{1348., 1732.}	14.3588	0.0573209	newly 5 weeks spec	237.	426.79	{364., 471.}	80.0802	0.253393	newly 5 weeks cumul	1901.	2443.74	{2095., 2684.}	28.5786	0.107508	newly 53 weeks cumul	2836.	4891.52	{4043., 5269.}	72.4795	0.23468
45	newly 3 weeks spec	487.	705.38	{638., 754.}	44.8419	0.159865	newly 3 weeks cumul	1897.	2317.99	{2157., 2435.}	22.3727	0.0871776	newly 5 weeks spec	273.	508.48	{467., 561.}	86.2564	0.268537	newly 5 weeks cumul	2573.	3441.25	{3162., 3602.}	33.848	0.126038	newly 56 weeks cumul	3089.	5465.15	{5112., 5752.}	76.923	0.246912
46	newly 3 weeks spec	405.	519.12	{429., 590.}	28.7901	0.107598	newly 3 weeks cumul	1271.	1561.59	{1298., 1760.}	23.7931	0.0908559	newly 5 weeks spec	319.	455.14	{355., 532.}	43.2226	0.152327	newly 5 weeks cumul	1984.	2511.56	{2063., 2813.}	27.3579	0.102799	newly 55 weeks cumul	2681.	5201.7	{4322., 5887.}	94.0224	0.28424
47	newly 3 weeks spec	463.	491.94	{373., 578.}	14.4622	0.0609917	newly 3 weeks cumul	1416.	1488.24	{1205., 1752.}	12.0918	0.0507493	newly 5 weeks spec	259.	423.01	{306., 504.}	63.6641	0.207098	newly 5 weeks cumul	2039.	2370.81	{1865., 2751.}	19.9671	0.0782382	newly 56 weeks cumul	2825.	4783.39	{3293., 5598.}	69.6067	0.222085
48	newly 3 weeks spec	614.	523.35	{484., 558.}	14.7638	0.0699806	newly 3 weeks cumul	1986.	1642.61	{1526., 1729.}	17.2905	0.0829207	newly 5 weeks spec	377.	439.97	{396., 476.}	16.9788	0.0669467	newly 5 weeks cumul	2819.	2571.79	{2388., 2692.}	8.76942	0.0403054	newly 53 weeks cumul	3973.	5007.24	{4614., 5481.}	26.0317	0.0994607
49	newly 3 weeks spec	412.	557.68	{465., 632.}	35.5583	0.129889	newly 3 weeks cumul	1467.	1709.16	{1467., 1933.}	17.741	0.0699775	newly 5 weeks spec	214.	475.55	{405., 546.}	122.22	0.34401	newly 5 weeks cumul	2027.	2710.8	{2426., 3089.}	33.853	0.124793	newly 55 weeks cumul	2605.	5274.4	{4616., 5846.}	102.472	0.304318
50	newly 3 weeks spec	434.	804.71	{737., 860.}	85.4171	0.266439	newly 3 weeks cumul	2003.	2889.97	{2694., 3058.}	44.2821	0.158335	newly 5 weeks spec	156.	498.1	{455., 539.}	219.295	0.500722	newly 5 weeks cumul	2450.	4026.1	{3800., 4258.}	64.3306	0.214653	newly 60 weeks cumul	2707.	5520.39	{5169., 5882.}	103.93	0.307574
